# Probiotics Exhibit Strain-Specific Protective Effects in T84 Cells Challenged With *Clostridioides difficile*-Infected Fecal Water

**DOI:** 10.3389/fmicb.2021.698638

**Published:** 2022-01-26

**Authors:** Mohd Baasir Gaisawat, Silvia Lopez-Escalera, Chad W. MacPherson, Michèle M. Iskandar, Thomas A. Tompkins, Stan Kubow

**Affiliations:** ^1^School of Human Nutrition, McGill University, Montréal, QC, Canada; ^2^Department of Nutrigenomics, Wageningen University, Wageningen, Netherlands; ^3^Rosell Institute for Microbiome and Probiotics, Montréal, QC, Canada

**Keywords:** probiotics, T84 cells, *C. difficile*, inflammation, cytokines, *cytotoxicity*, fecal water, gastrointestinal model (GI)

## Abstract

*Clostridioides difficile* infection (CDI) is frequently associated with intestinal injury and mucosal barrier dysfunction, leading to an inflammatory response involving neutrophil localization and upregulation of pro-inflammatory cytokines. The severity of clinical manifestations is associated with the extent of the immune response, which requires mitigation for better clinical management. Probiotics could play a protective role in this disorder due to their immunomodulatory ability in gastrointestinal disorders. We assessed five single-strain and three multi-strain probiotics for their ability to modulate CDI fecal water (FW)-induced effects on T84 cells. The CDI-FW significantly (*p* < 0.05) decreased T84 cell viability. The CDI-FW-exposed cells also exhibited increased pro-inflammatory cytokine production as characterized by interleukin (IL)-8, C-X-C motif chemokine 5, macrophage inhibitory factor (MIF), IL-32, and tumor necrosis factor (TNF) ligand superfamily member 8. Probiotics were associated with strain-specific attenuation of the CDI-FW mediated effects, whereby *Saccharomyces boulardii* CNCM I-1079 and *Lacticaseibacillus rhamnosus* R0011 were most effective in reducing pro-inflammatory cytokine production and in increasing T84 cell viability. ProtecFlor™, *Lactobacillus helveticus* R0052, and *Bifidobacterium longum* R0175 showed moderate effectiveness, and *L. rhamnosus* GG R0343 along with the two other multi-strain combinations were the least effective. Overall, the findings showed that probiotic strains possess the capability to modulate the CDI-mediated inflammatory response in the gut lumen.

## Introduction

*Clostridioides* (formerly *Clostridium difficile*) infection (CDI) is a toxin-mediated intestinal disease that is the most frequently identified cause of healthcare-associated infectious diarrhea (Awad et al., 2014). The development of CDI is strongly associated with the alteration of bile acid metabolism and disruption of gut microbiota through the use of broad-spectrum antibiotics, allowing for optimal conditions for spore germination and subsequent colonization of the gut lumen (Shen, 2012). The clinical manifestations of CDI range in severity from mild diarrhea to life-threatening pseudomembranous colitis (Rupnik et al., 2009). The pathogenesis of CDI is strongly associated with the production of enterotoxin A (TcdA) and enterotoxin B and the presence of other virulence factors such as S-layer proteins and flagellin (Thelestam and Chaves-Olarte, 2000; Ausiello et al., 2006; Stevenson et al., 2015). These factors enable *C. difficile* to induce an acute inflammatory response in intestinal cells, resulting in neutrophil activation and recruitment that ultimately lead to intestinal epithelial damage (Chaves-Olarte et al., 1997; Brito et al., 2002). Thus, the clinical manifestations of CDI can be attributed to the various *C. difficile* virulence factors acting in conjunction with the host immune response (Kelly and Kyne, 2011). In this respect, it is noteworthy that there is growing evidence suggesting that the host immune response is an important predictor of clinical severity and adverse outcomes in CDI patients (Kelly and Kyne, 2011; El Feghaly et al., 2013b; Yu et al., 2017)—for instance, fecal C-X-C motif chemokine 5 (CXCL5) and interleukin (IL)-8, not bacterial burden, were correlated with clinical severity in CDI patients (El Feghaly et al., 2013a). Furthermore, a recent study on human and murine CDI found that assessing multiple inflammatory serum markers can better predict adverse outcomes as compared to the currently used methods of predicting CDI mortality (Dieterle et al., 2020). Specifically, inflammatory markers such as procalcitonin and hepatocyte growth factor were found to be the best predictor for disease severity, and IL-8, CXCL5, CXCL10, and IL-2Rα were the best predictors of 30-day mortality (Dieterle et al., 2020). Accordingly, the mitigation of CDI-mediated inflammation could play an important role in regulating the host immune response, leading to better management of CDI outcomes (Kelly and Kyne, 2011).

One of the most widely used therapeutic strategies to modulate the host immune response in gastrointestinal disorders, including CDI, has been through probiotic supplementation (Andrade et al., 2015). Probiotics have been demonstrated to confer a wide variety of beneficial effects in the management of gastrointestinal (GI) disorders, including enhancement of mucosal barrier function (Generoso et al., 2010; Ueno et al., 2011), counteracting infections by producing antimicrobial compounds and stimulation of host antimicrobial defense pathways (McFarland et al., 1994; Collado et al., 2007; Marteau et al., 2007), modulating immune function (Andrade et al., 2015), and attenuating clinical manifestations (Sartor, 2005; Boirivant and Strober, 2007; Marteau et al., 2007; Elmadfa et al., 2010). The majority of probiotics used commercially are from the *Lactobacilli*, *Bifidobacteria*, and yeast (*Saccharomyces*) groups. Although research has demonstrated the potential of probiotics to act as immunomodulators, their effects are largely seen to be strain specific, and much is yet to be elucidated on their mechanisms of action. *Saccharomyces boulardii* has demonstrated to stimulate intestinal anti-toxin immunoglobulin A (Qamar et al., 2001), inhibit IL-8 production, activate mitogen-activated protein (MAP) kinases (Chen et al., 2006), and produce soluble anti-inflammatory factors that inhibit nuclear factor (NF)-κB-mediated IL-8 gene expression (Sougioultzis et al., 2006). Similarly, experimental evidence demonstrates the ability of several *Lactobacilli* spp. and *Bifidobacteria* spp. to modulate immune activity primarily through the secretion of a variety of molecules that directly or indirectly promote the inactivation of NF-κB signaling pathways (MacPherson et al., 2014; Jeffrey et al., 2020). *Lacticaseibacillus rhamnosus* GG (previously known as *Lactobacillus rhamnosus*) was shown to prevent cytokine-induced apoptosis in several intestinal epithelial cell models (Yan and Polk, 2002), whereas *L. rhamnosus* L34 and *L. casei* L39 were demonstrated to modulate CDI-mediated inflammation by decreasing IL-8 expression and the inactivation of NF-κB (Boonma et al., 2014). Moreover, *L. rhamnosus* R0011 and *Lactobacillus helveticus* R0389 were shown to secrete bioactive molecules capable of downregulating IL-8 production in HT-29 epithelial cells (Jeffrey et al., 2018, 2020).

Despite the promising role of probiotics to modulate the host immune response to bacterial pathogens and stressors, much remains to be elucidated concerning their ability to mitigate the CDI-associated inflammatory cascade. In the present study, we utilized an *in vitro* GI model to simulate CDI, defined as diarrheal stool from an adult patient that was positive for one or both enterotoxins A and B (Gaisawat et al., 2019b; Shoaei et al., 2019). We assessed the effects of CDI fecal water (FW) from this model on colonic adenocarcinoma-derived T84 epithelial cell viability and immune marker production. Additionally, the effects of various probiotic-supplemented CDI-FW were assessed for efficacy to protect against potential CDI-mediated effects.

## Materials and Methods

### Probiotic Treatments

In this study, eight different probiotic treatments were assessed for their ability to cause changes in *C. difficile*-infected microbiota. Five of the treatments were single-strain probiotics, whereas the remaining three were various combinations of single-strain probiotics (Table 1).

**TABLE 1:** Probiotic treatments and dosage.

Probiotic	Code	Dosage (cfu/vessel)
*L. rhamnosus* R0011	R0011	1 × 10^9^
*L. helveticus* R0052	R0052	1 × 10^9^
*L. rhamnosus* GG R0343	R0343	1 × 10^9^
*S. boulardii* CNCM I-1079	SB	1 × 10^9^
*B. longum* R0175	R0175	1 × 10^9^
ProtecFlor™ (combination of R0011, R0052, SB, and R0175)	PROTO	1 × 10^9^
Combination 2 (R0011, R0052, and R0175)	R0011 + R0052 + R0175	1 × 10^9^ of each strain
Combination 3 (R0343 and SB)	R0343 + SB	10 × 10^9^ of R0343 and 5 × 10^9^ of SB

All probiotics were acquired from Lallemand Health Solutions Inc. (Montreal, QC, Canada) and stored at –20°C until use. For inoculation in batch culture fermentation, the probiotics were mixed in sterile phosphate-buffered saline (PBS). PBS alone was used as the negative control (blank).

### Simulation of Gastrointestinal Conditions

Batch culture fermentation was performed to simulate the conditions of *C. difficile* infection using a computer-controlled GI model consisting of several independent anaerobic fermentation vessels run under physiological conditions as described previously in detail (Gaisawat et al., 2019a). Briefly, 100 ml of filter-sterilized GI food, previously optimized by Molly et al. (1994) consisting of arabinogalactan, pectin, xylan, potato starch, glucose, yeast extract, peptone, mucin, and cysteine powders (Sigma Aldrich, St. Louis, MO, United States), was added to each vessel. This was followed by sequential enzymatic digestion by the addition of α-amylase (pH 7.0 for 15 min), followed by pepsin (pH 2.0 for 1.5 h), and, finally, by pancreatic juice (12 g/L NaHCO_3_, 6 g/L bile extract, and 0.9 g/L pancreatin; pH 8.0 for 2 h). After completion of digestion, 50 ml of fecal slurry was inoculated (*T* = 0 h) to simulate the gut microbiota. A fecal sample obtained from a healthy male adult donor with no history of GI disorders and no antibiotic use in the past 6 months was used to make normal fecal slurry, whereas CDI fecal slurry was prepared using a 1:10 v/v fecal inoculation from a commercially obtained *C. difficile* fecal sample (male adult with stool positive for *C. difficile* toxins A and B; BioIVT, Westbury, NY, United States). Premixed probiotic treatments or blank was subsequently added to each vessel, followed by anerobic fermentation at 6.3 ± 0.3 pH for 24 h. The samples taken at each time point were centrifuged at 2,000 *g* for 10 min and stored at –80°C.

### Sample Preparation for Cell Culture

The samples collected from the batch fermentation at *T* = 0 h and *T* = 24 h were further centrifuged at 13,000 *g* for 20 min, and the supernatant (hereinafter referred to as FW) was collected and filter-sterilized with a 0.22-μm syringe-driven filter (Fisher Scientific, Ottawa, ON, Canada). FW from each fermentation replicate was pooled before storage at –80°C until administration to the cells. The samples collected at *T* = 0 h from the probiotic blank vessels were considered as the controls for normal and CDI-fecal water, respectively.

### Cell Culture

The human colonic adenocarcinoma cell line T84 was obtained from the American Type Culture Collection (Burlington, ON, Canada) and cultured according to the procedures of the company. Briefly, the T84 cells were cultured with Dulbecco’s modified Eagle’s medium nutrient mixture (DMEM:F12) supplemented with 5% fetal bovine serum (FBS) in 75-cm^2^ T-flasks until 80% confluency was reached. The cells were incubated at 37°C with 5% CO_2_ and 90% humidity, and the medium was renewed every second day with the appropriate subcultivation ratio of 1:2–1:4 performed on a bi-weekly basis. Three separate cell passages (above passage number 15) were maintained concurrently for the treatment experiment.

For the experiment, T84 cells were detached at 80% confluency with 0.25% trypsin-EDTA solution for 5–10 min, subsequently seeded at a density of 2 × 10^6^ cells/well onto 24-well plates (Costar^®^ 24-well TC-treated Multiple Well Plates; Corning, NY, United States), and grown overnight under the same incubation conditions as described above. Prior to starting the treatments, the confluency of the monolayer was checked under a microscope. A dose–response experiment was previously carried out in order to determine the optimal dose of the normal FW blank *T* = 0 h on the T84 cells with a minimum effect to their viability for an 8-h incubation period (> 90% viability). As a result of this, 30% (v/v) FW in cell culture medium was deemed appropriate for further use (data not shown). After monolayer formation in the plates, the cell media were discarded, followed by the addition of 1% FBS-supplemented fresh medium (1,000 μl/well) along with filtered FW (500 μl/well). All treatments were added in triplicate for each cell passage number and subsequently incubated for a period of 8 h. After incubation, the supernatant from each well was collected, centrifuged at 13,000 *g* for 10 min, and stored at –20°C for further analyses. Cell viability was determined for the remaining cells in the 24-well plate.

### 3-(4,5-Dimethylthiazol-2-yl)-2,5-Diphenyltetrazolium Bromide Assay

The 3-(4,5-dimethylthiazol-2-yl)-2,5 diphenyltretrazolium bromide (MTT) reduction assay was performed as a measure of cellular viability. The assay measured the ability of viable cells to convert the pale yellow MTT reagent to a purple-colored crystalline formazan through nicotinamide adenine dinucleotide phosphate-dependent cellular oxidoreductase enzymes (Berridge and Tan, 1993). Briefly, 500 μl of MTT solution (0.5 mg/ml MTT in phenol red-free DMEM:F12 medium) was added to each well on a plate after the removal of supernatants from the experiment, followed by incubation for a period of 3 h. After incubation, the supernatant was discarded, and 500 μl acidified isopropyl alcohol (0.4 N HCl) was added to each well and allowed to react for 5 min at room temperature. The contents of each well were transferred to a 96-well plate and read spectrophotometrically at λ = 570 nm. The results were expressed as a percentage of untreated control cells.

### Cytokine and Chemokine Determination

The detection of various cytokines and chemokines following the exposure of T84 cells to the treatments was determined by multiplex assays. Bio-Plex Pro™ Human Chemokine 40-plex Panel (cat. no. 171AK99MR2, Bio-Rad, Hercules, CA, United States) was used to detect chemokine expression, and Bio-Plex Pro™ Human Inflammation 37-plex Panel (cat. no. 171AL001M, Bio-Rad, Hercules, CA, United States) was used to determine inflammatory cytokine expression. Each assay was performed according to the instructions of the manufacturer. The samples tested in these kits included the supernatants collected from T84 cells exposed to the normal FW blank (*T* = 0 h and *T* = 24 h) and each of the *C. difficile*-infected FW treatments (blank + 8 probiotic interventions: *T* = 0 h and *T* = 24 h). Each sample treatment was tested using three biological replicates. Standard curves for each cytokine and chemokine were generated in duplicate using serial dilutions of the premixed lyophilized standards provided in the kits. Data was acquired with the help of a Bio-Plex 200 instrument (Bio-Rad, Hercules, CA, United States) and analyzed by the Bio-Plex Manager software for concentration in range (pg/ml) (v 4.1, Bio-Rad, Hercules, CA, United States). Quality checks were done for each chemokine and cytokine using the respective working range and limit of detection data provided in the product lot sheets for each kit. The results for each marker were expressed as picogram per milliliter.

### Statistical Analysis

All data are reported as means ± standard error of mean. Normality was assessed on original data sets and achieved with log transformations where necessary to align with statistical assumptions. Data for cell viability assays after the treatment of cells with fecal water collected at 24 h were analyzed using one-way ANOVA for each fecal type using treatment (9 levels) as a factor, followed by Dunnett’s *post-hoc* analysis to compare with the control. Data for cytokine analyses were assessed using two-way ANOVA for CDI-FW using time (2 levels) and treatment (9 levels), followed by Tukey’s *post-hoc* analysis. When significant interactions between time and treatment were observed, the means of each time point within a fecal type were individually compared for significant differences within the fecal type. Statistical significance was set at *p* < 0.05. All data analyses and visualizations were performed using JMP v14.4 (SAS Institute, Cary, NC, United States), with the exception of the heat map, which was generated with GraphPad Prism (v 7.04, GraphPad Software Inc., San Diego, CA, United States).

## Results and Discussion

*C. difficile* toxins have been extensively studied in various intestinal cell cultures to elucidate their cytotoxic effects and their ability to induce inflammatory cytokines (Flegel et al., 1991; Branka et al., 1997; Thelestam and Chaves-Olarte, 2000; Ausiello et al., 2006; El Feghaly et al., 2013a; Yu et al., 2017). The present investigation is the first to assess the cytotoxic and proinflammatory effects of *C. difficile*-infected fecal water such as that obtained from a simulated human GI digestion model. This method allows for a more holistic approach to study CDI fecal sample assessment involving human gut epithelial cells rather than *C. difficile* cultures or purified toxins (Canny et al., 2001; Johal et al., 2004). In the present study, we assessed the ability of *C. difficile*-infected microbiota to cause changes to T84 cell viability and cytokine expression following exposure of these cells to FW collected from the batch fermentation at *T* = 0 h and *T* = 24 h.

### Cell Viability and Cytotoxicity

**FIGURE 1:**
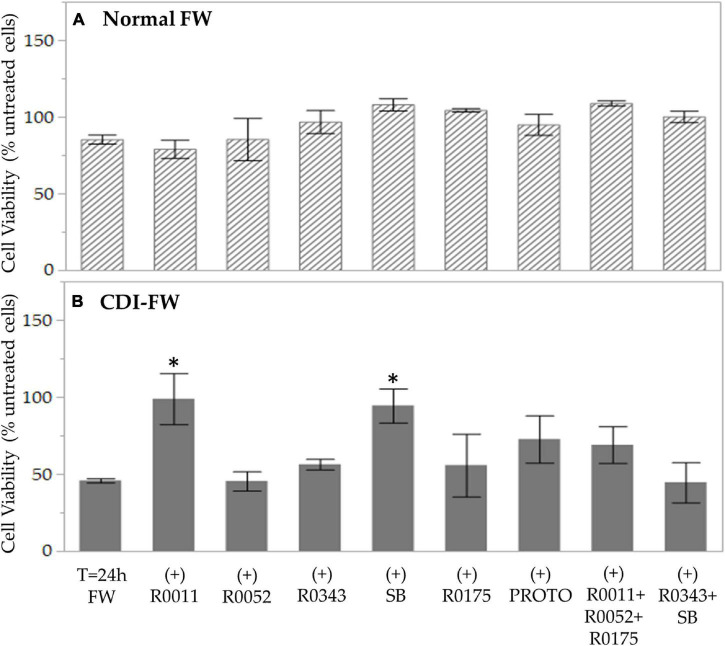
Cell viability following the exposure of T84 cells to fecal water (FW) treatments as measured by the 3-(4,5-dimethylthiazol-2-yl)-2,5-diphenyltetrazolium bromide assay. **(A)** Cells treated with normal FW and **(B)** cells treated with *Clostridioides difficile*-infected (CDI) FW. Values are shown as mean ± SEM. The symbol * represents significant differences between the means of treatments (*p* < 0.05). R0011 = *L. rhamnosus* R0011; R0052 = *L. helveticus* R0052; R0343 = *L. rhamnosus* GG R0343; SB = *S. boulardii* CNCM I-1079; R0175 = *B. longum* R0175; PROTO = ProtecFlor™; R0011+ R0052+R0175 = combination of *L. rhamnosus* R0011, *L. helveticus* R0052 and *B. longum* R0175; R0343+SB = combination of *L. rhamnosus* GG R0343 and *S. boulardii* CNCM I-1079.

The MTT assay, a well-documented method of assessment of cell survival and growth (Korzeniewski and Callewaert, 1983; Morgan, 1998), was performed to study the effect of FW on T84 cell viability. In the results from the assay, exposure to normal FW did not show a significant change in T84 cell viability. On the other hand, T84 exposure to CDI-FW resulted in 2.1-fold decrease in cell viability (Figure 1). In the cells treated with CDI-FW containing the probiotic treatments *L. rhamnosus* R0011 and *S. boulardii* CNCM I-1079, cell viability was significantly (*p* < 0.05) higher in comparison to the CDI-FW blank sample (Figure 1). Interestingly, the cell viability of *L. rhamnosus* R0011 and *S. boulardii* CNCM I-1079 CDI-FW-treated cells was similar to that of untreated cells, indicating a protective effect of these two probiotic strains in CDI-FW.

The results from the MTT assay are in concordance with previously documented studies showing the cytotoxic potency of *C. difficile* and its toxins (Mahida et al., 1996; Branka et al., 1997; Thelestam and Chaves-Olarte, 2000; Brito et al., 2002; Chumbler et al., 2012). Notably, in our study, this effect was observed by exposing the cells to CDI-FW obtained from our simulated GI digestion model of CDI microbiota, as opposed to a previous work using either cultured *C. difficile* strains or their purified toxins. Fecal water has been previously demonstrated to possess some cytotoxic activity (Pearson et al., 2009; Federici et al., 2017), which, in the case of CDI-FW, could have been further exacerbated by the presence of its enterotoxins and secreted products such as proteolytic and hydrolytic enzymes (Vedantam et al., 2012). This is further supported by previous studies by our group showing that CDI-FW demonstrates dysregulation in gut metabolic function and antioxidant status, potentially leading to a cytotoxic environment (Gaisawat et al., 2019a,b).

Probiotic-supplemented CDI-FW showed, to an extent, the ability to counteract some of the cytotoxic effects of the CDI-FW. In particular, the probiotic supplements R0011 and SB demonstrated a significant (*p* < 0.05) increase in T84 cell viability, resulting in values like those observed in T84 cells exposed to normal FW (Figure 1). These results indicate that probiotics potentially act in a strain-specific manner to counteract the cytotoxicity of *C. difficile*, a phenomenon that has been previously demonstrated (Kekkonen et al., 2008). In CDI, *S. boulardii* is mainly thought to act through immune system regulation (Qamar et al., 2001; Stier and Bischoff, 2016) and the production of anti-toxin proteases, which could counteract CDI-mediated pathophysiology (Castagliuolo et al., 1999). With regards to *Lactobacilli* spp. and *Bifidobacterium* spp., *in vitro* evidence is limited; however, it is suggested that these probiotics might counteract CDI-mediated effects by preventing *C. difficile* adhesion (Trejo et al., 2006; Lee et al., 2013), maintaining intestinal barrier integrity (Vanderpool et al., 2008), and modulating the host immune response (Ng et al., 2008).

### Immune Response of T84 Cells Following Fecal Water Exposure

*C. difficile* exposure *in vitro* has been previously shown to be associated with intestinal tissue damage followed by a robust immune response that upregulates proinflammatory cytokines and recruits neutrophils, further leading to an acute inflammatory response (Sun et al., 2010). Furthermore, it has been noted that monitoring the immune response in patients with CDI may be a more suitable marker for disease severity rather than bacterial burden (El Feghaly et al., 2013a).

**FIGURE 2:**
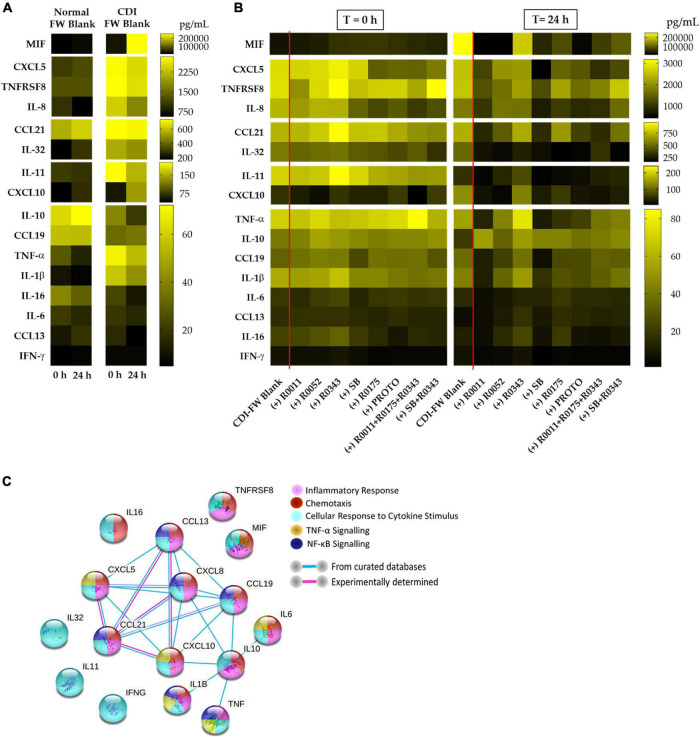
Cytokine and chemokine profiles from T84 cells exposed to the different fecal water (FW) treatments (collected at *T* = 0 h and *T* = 24 h from the batch fermentation) for a period of 8 h. **(A)** Cytokine and chemokine profiles in normal FW blank and CDI-FW blank. **(B)** Cytokine and chemokine profiles in CDI-FW supplemented with probiotic treatments. Data shown are the mean cytokine/chemokine production (picograms per milliliter; *n* = 4). **(C)** STRING v 11.0 analysis showing functional links between each of the different cytokines/chemokines produced. R0011 = *L. rhamnosus* R0011; R0052 = *L. helveticus* R0052; R0343 = *L. rhamnosus* GG R0343; SB = *S. boulardii* CNCM I-1079; R0175 = *B. longum* R0175; PROTO = ProtecFlor™; R0011+ R0052+R0175 = combination of *L. rhamnosus* R0011, *L. helveticus* R0052 and *B. longum* R0175; R0343+SB = combination of *L. rhamnosus* GG R0343 and *S. boulardii* CNCM I-1079.

In our study, we chose to evaluate a wide range of chemokines and cytokines as a tool to assess the host immune response of T84 cells to CDI-FW. These molecules were quantified using two multiplex assay kits comprising of a 40-plex chemokine panel and a 37-plex inflammatory cytokine panel (Bio-Rad, Hercules, CA, United States). The results from the multiplex assays, summarized in the heat map (Figure 2), showed an increased production of a host of chemokines and inflammatory cytokines by the T84 cells following exposure to CDI-FW. These molecules primarily include interleukins 8, 11, and 32, CXCL5, tumor necrosis factor (TNF) surface receptor 8, macrophage inhibitory factor (MIF), and C-C motif chemokine ligand 21 (CCL21) among others. Notably, CDI-FW was shown to upregulate almost all of these chemokines and cytokines in comparison to normal FW, except for the anti-inflammatory IL-10. Most of these molecules have been previously associated with inflammatory pathways, with chemotaxis, and in cytokine signaling of the TNF-α and NF-κB pathways (Thelen and Uguccioni, 2016). Importantly, the results from the multiplex assays showed anti-inflammatory effects of probiotic supplementation in CDI-FW at *T* = 24 h, whereby several probiotic treatments showed attenuation in the production of several chemokines and cytokines (Figure 2).

### Chemokine Expression

IL-8 and CXCL5 followed by CCL21 were the prominent chemokines expressed in the cultured T84 cells following exposure to CDI-FW. Both IL-8 (also known as CXCL8) and CXCL5 hail from the same family of CXC chemokines that are involved in the activation of the CXCR2 receptor, ultimately leading to chemotaxis of neutrophils and inducing innate immunity (Murphy, 2007). CCL21, on the other hand, has been shown to play a role in the chemotaxis of leukocytes, such as T cells (Thelen and Uguccioni, 2016).

**FIGURE 3:**
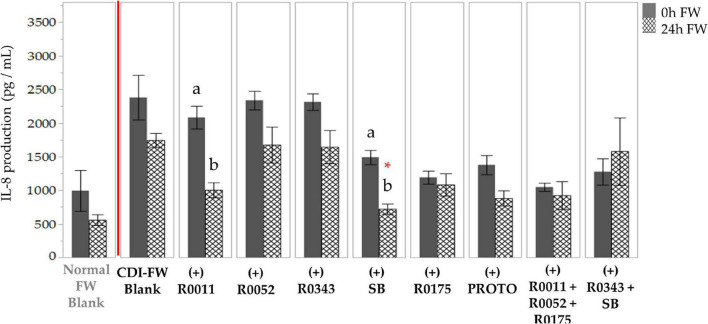
Detection of interleukin-8 production following the exposure of T84 cells to *Clostridioides difficile*-infected (CDI) fecal water (FW) treatments. 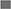, cells treated with FW collected at *T* = 0 h; 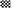, cells treated with FW collected at *T* = 24 h. Means at time points within treatments not sharing a common letter are significantly different from each other (*p* < 0.05). The symbol * represents significant differences (*p* < 0.05) between treatment and CDI-FW blank at *T* = 24 h. R0011 = *L. rhamnosus* R0011; R0052 = *L. helveticus* R0052; R0343 = *L. rhamnosus* GG R0343; SB = *S. boulardii* CNCM I-1079; R0175 = *B. longum* R0175; PROTO = ProtecFlor™; R0011+ R0052+R0175 = combination of *L. rhamnosus* R0011, *L. helveticus* R0052 and *B. longum* R0175; R0343+SB = combination of *L. rhamnosus* GG R0343 and *S. boulardii* CNCM I-1079.

The results of IL-8 production by T84 cells exposed to CDI-FW when compared to normal FW at *T* = 24 h showed a 3.1-fold increase (Supplementary Table 1). In CDI-FW treatments, the two-way ANOVA results showed significant (*p* < 0.05) effects of treatment, time, and interaction. Therefore, the mean IL-8 values from cells exposed to CDI-FW collected at the two time points (*T* = 0 h and *T* = 24 h) were compared to their corresponding time points within the blank to assess for any differences (Figure 3). The probiotic SB was the only treatment that showed a significant (*p* < 0.05) decrease in IL-8 production when compared to the control (*T* = 24 h). R0011 showed a significant (*p* < 0.05) decrease across time but did not show statistical significance when compared to the control at the corresponding time point. No other probiotic-supplemented CDI-FW showed a statistical difference when compared to the blank.

**FIGURE 4:**
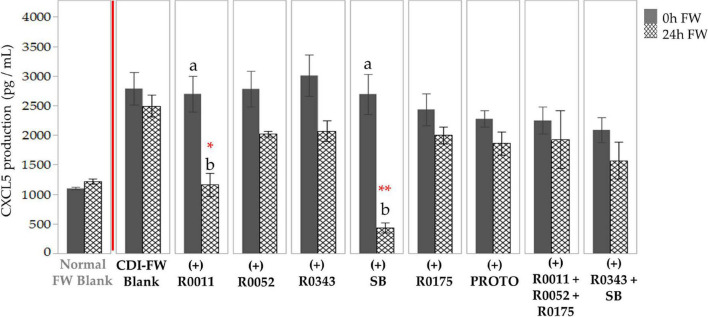
Detection of C-X-C motif chemokine 5 production following the exposure of T84 cells to *Clostridioides difficile*-infected (CDI) fecal water (FW) treatments. 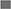, cells treated with FW collected at *T* = 0 h; 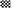, cells treated with FW collected at *T* = 24 h. Values are shown as mean ± SEM. Means at time points within treatments without a common letter are significantly different (*p* < 0.05). The symbol * represents significant differences (*p < 0.05; **p < 0.01) between treatment and CDI-FW blank at *T* = 24 h. R0011 = *L. rhamnosus* R0011; R0052 = *L. helveticus* R0052; R0343 = *L. rhamnosus* GG R0343; SB = *S. boulardii* CNCM I-1079; R0175 = *B. longum* R0175; PROTO = ProtecFlor™; R0011+ R0052+R0175 = combination of *L. rhamnosus* R0011, *L. helveticus* R0052 and *B. longum* R0175; R0343+SB = combination of *L. rhamnosus* GG R0343 and *S. boulardii* CNCM I-1079.

CDI-FW also showed an upregulation in CXCL5 production in T84 cells at *T* = 24 h, showing a twofold increase as compared to the normal FW treatment (Supplementary Table 1). The two-way ANOVA analysis of the data for cells exposed to CDI-FW treatments showed significant (*p* < 0.05) main effects of treatment, time, and their interaction. CDI-FW supplemented with R0011 and SB, respectively, were the only treatments that showed a significant (*p* < 0.05) decrease in CXCL5 production when compared to the blank at *T* = 24 h (Figure 4).

In terms of CCL21, exposure of T84 cells to CDI-FW did not result in increased production when compared to normal FW. The findings demonstrated, however, a significant (*p* < 0.05) decrease in CDI-FW treated with probiotics, indicating a potential role of R0011, R0052, SB, PROTO, and combination 2 (R0011 + R0052 + R0175) in modulating CCL21 production (Supplementary Figure 1).

### Cytokine Expression

In addition to chemokine production, T84 cells challenged with CDI-FW were associated with the increased production of several inflammatory cytokines, such as MIF, TNFRSF8, and IL-32. MIF is an inflammatory cytokine that has been associated with the host immune response to infectious pathogens such as CDI (Oddo et al., 2005; Jose et al., 2018). TNFRSF8, also referred to as CD30, has been previously shown to mediate signal transduction, leading to the activation of NF-κB pathway (Buchan and Al-Shamkhani, 2012). Similarly, IL-32 has also been previously shown to induce cytokine signaling pathways, leading to the activation of NF-κB, TNF-α, and IL-8 (Kim et al., 2005).

**FIGURE 5:**
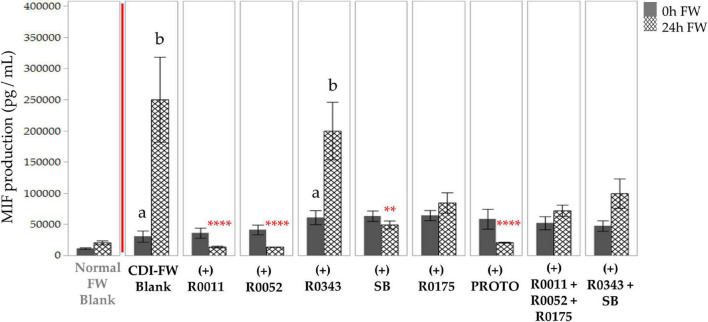
Detection of macrophage inhibitory factor production following the exposure of T84 cells to *Clostridioides difficile*-infected (CDI) fecal water (FW) treatments. 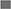, cells treated with FW collected at *T* = 0 h; 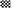, cells treated with FW collected at *T* = 24 h. Values are shown as mean ± SEM. Means at time points within treatments not sharing a common letter are significantly different from each other (*p* < 0.05). The symbol * represents significant differences (**p < 0.01; ****p < 0.0001) between treatment and CDI-FW blank at *T* = 24 h. R0011 = *L. rhamnosus* R0011; R0052 = *L. helveticus* R0052; R0343 = *L. rhamnosus* GG R0343; SB = *S. boulardii* CNCM I-1079; R0175 = *B. longum* R0175; PROTO = ProtecFlor™; R0011+ R0052+R0175 = combination of *L. rhamnosus* R0011, *L. helveticus* R0052 and *B. longum* R0175; R0343+SB = combination of *L. rhamnosus* GG R0343 and *S. boulardii* CNCM I-1079.

CDI-FW-challenged T84 cells showed an initial 2.7-fold difference in MIF production when compared to normal FW at *T* = 0 h. At *T* = 24 h, however, this difference in MIF production was an increase of 12.1-fold (Figure 5). The significant (*p* < 0.05) increase in MIF production was attenuated when the cells were exposed to probiotic-treated CDI-FW. The probiotic treatments R0011, R0052, SB, and PROTO showed a significant (*p* < 0.05) decrease in MIF production at *T* = 24 h when compared to the CDI-FW blank. A tendency for this attenuation was also observed with the probiotics R0175, combination 2, and combination 3, although this did not reach statistical significance. CDI-FW supplemented with R0343 was the only probiotic treatment that resulted in a significant (*p* < 0.05) increase in MIF over time, indicating no effect of R0343 on MIF production in CDI-FW.

**FIGURE 6:**
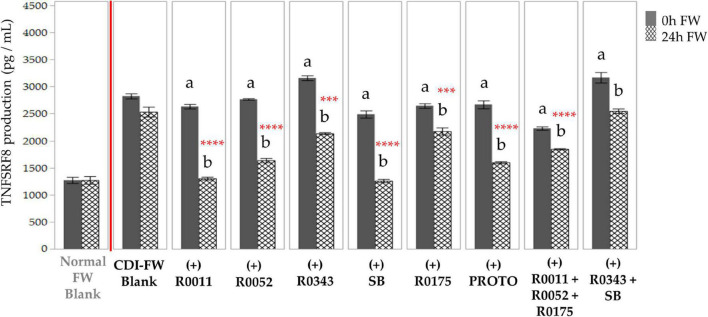
Detection of tumor necrosis factor receptor superfamily member 8 production following the exposure of T84 cells with *Clostridioides difficile*-infected (CDI) fecal water (FW) treatments as measured by multiplex assay. 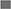, cells treated with FW collected at *T* = 0 h; 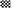, cells treated with FW collected at *T* = 24 h. Values are shown as mean ± SEM. Means at time points within treatments without a common letter are significantly different from each other (*p* < 0.05). The symbol * represents significant differences (***p < 0.001; ****p < 0.0001) between treatment and CDI-FW blank at *T* = 24 h. R0011 = *L. rhamnosus* R0011; R0052 = *L. helveticus* R0052; R0343 = *L. rhamnosus* GG R0343; SB = *S. boulardii* CNCM I-1079; R0175 = *B. longum* R0175; PROTO = ProtecFlor™; R0011+ R0052+R0175 = combination of *L. rhamnosus* R0011, *L. helveticus* R0052 and *B. longum* R0175; R0343+SB = combination of *L. rhamnosus* GG R0343 and *S. boulardii* CNCM I-1079.

T84 cells exposed to CDI-FW also resulted in a twofold increase of TNFRSF8 production in comparison to normal FW at *T* = 24 h (Figure 6). Each of the probiotic treatments in CDI-FW, except for combination 3 (R0343 + SB), showed a significant (*p* < 0.05) decrease in TNFRSF8 production at *T* = 24 h when compared to the CDI-FW blank.

**FIGURE 7:**
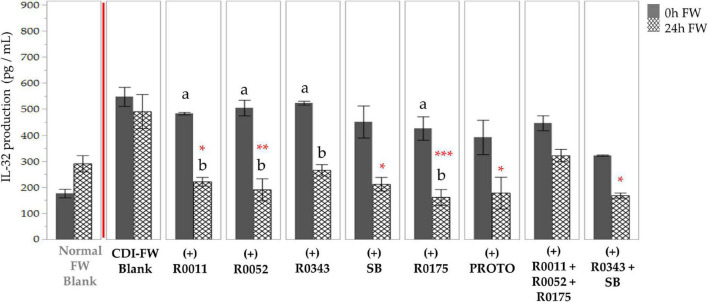
Detection of interleukin-32 production following the exposure of T84 cells with *Clostridioides difficile*-infected (CDI) fecal water (FW) treatments as measured by multiplex assay. 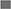, cells treated with FW collected at *T* = 0 h; 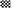, cells treated with FW collected at *T* = 24 h. Values are shown as mean ± SEM. Means at time points within treatments without a common letter are significantly different from each other (*p* < 0.05). The symbol * represents significant differences (*p < 0.05; **p < 0.01; ***p < 0.001) between treatment and CDI-FW blank at *T* = 24 h. R0011 = *L. rhamnosus* R0011; R0052 = *L. helveticus* R0052; R0343 = *L. rhamnosus* GG R0343; SB = *S. boulardii* CNCM I-1079; R0175 = *B. longum* R0175; PROTO = ProtecFlor™; R0011+ R0052+R0175 = combination of *L. rhamnosus* R0011, *L. helveticus* R0052 and *B. longum* R0175; R0343+SB = combination of *L. rhamnosus* GG R0343 and *S. boulardii* CNCM I-1079.

The data for IL-32 followed a similar trend, showing a 1.7-fold increase in the production of the cytokine when T84 cells were exposed to CDI-FW (*T* = 24 h) in comparison to normal FW. All probiotic-supplemented CDI-FW, apart from R0343 and combination 2 (R0011+ R0052 + R0175), showed a significant (*p* < 0.05) reduction in IL-32 production at *T* = 24 h (Figure 7).

Intriguingly, other commonly associated inflammatory cytokines in the context of CDI in *in vitro* studies, such as interferon (IFN)-γ, TNF-α, IL-6, or IL-1β (Usacheva et al., 2016), did not show a significant activation in our study, showing detection levels below 100 pg/ml (Supplementary Figures 3–11). This presumably could be due to the initial activation of IL-8 in T84 cells that is observed within a few hours of treatment exposure (Canny et al., 2006), whereas other cytokines, such as TNF-α, have been shown to be produced in significant amounts only after 48 h of TcdA exposure (Brito et al., 2002). Moreover, neutrophil activation and localization are seen to be the key hallmarks of CDI-mediated inflammation, possibly explaining the prominent upregulation of both IL-8 and CXCL5 (Kelly and Kyne, 2011). Despite the low concentrations observed, CDI-FW-exposed cells showed an increased production in key cytokines, TNF-α, and IL-1β (fourfold and sevenfold increase at *T* = 24 h, respectively; Supplementary Table 1). No overall effect of CDI-FW was observed on IL-6 and IFN-γ concentrations. The anti-inflammatory cytokine IL-10 showed a 0.3-fold decrease at *T* = 24 in the CDI-FW blank-exposed cells; however, this attenuation was restored to normal FW levels in probiotic-supplemented CDI-FW treatments (Supplementary Table 1). Additionally, IL-11, which plays a role in mediating an anti-inflammatory response through its interaction with the IL-6 signaling receptor (Harmegnies et al., 2003), was also observed to be produced in association with both CDI-FW and normal FW. In this case, however, CDI-FW exposure did not result in a significant difference in its production when compared to normal FW (Supplementary Figure 2), suggesting that this cytokine did not play a contributing factor in CDI-mediated inflammatory response.

Overall, the findings from chemokine and cytokine analyses showed the ability of CDI-FW to induce the production of inflammatory markers in T84 cells, namely, the chemokines IL-8 and CXCL5 and the cytokines MIF, TNFRSF8, and IL-32. In previous studies by El Feghaly et al. (2013b) and Dieterle et al. (2020) increased levels of IL-8 and CXCL5 were characteristic of the immune profiles of CDI patients and were key in predicting mortality in those patients. Our findings further reiterate the association of IL-8 and CXCL5 with CDI-mediated effects in the gut mucosa. The results from our cytokine analyses, however, demonstrated the presence of cytokines such as TNFRSF8, IL-32, and MIF that have been sparsely documented with respect to CDI. The role of MIF in the intestinal lumen is thought to be multifaceted, where several *in vitro* studies have demonstrated its ability to maintain epithelial barrier function and integrity by modulating the epithelial tight-junction proteins (Vujicic et al., 2018). Moreover, MIF has also been associated with several other roles, such as inhibition of cellular apoptosis by modulating MAP kinase signaling (Roger et al., 2013), eradication of gram-negative pathogens through macrophage action (Roger et al., 2013), and regulation of the magnitude of inflammatory response *via* glucocorticoid modulation (Donnelly and Baugh, 2006). With regard to CDI, however, the only experimental evidence to date elaborating the role of MIF is a study by Jose et al. (2018), which showed that, in a mouse model of CDI, systemic MIF was significantly upregulated, the neutralization of which led to a decrease in tissue inflammation, reduction in diarrhea, and increased survival. To our knowledge, the role of TNFSRF8 and IL-32 in CDI-associated inflammation has not been examined previously. Their role in CDI could be linked to their subsequent activation of the NF-κB pathway, which leads to the activation of cytokines, such as TNF-α, IL-6, IL-1β, and IL-8, all of which are more often tested for and associated with CDI (Limsrivilai et al., 2018).

Importantly, the results from the present study show the ability of several single-strain and multi-strain probiotic supplements to protect against CDI-FW-mediated inflammatory mediator production (Figure 2). The probiotic treatments showed varying effects on each of the cytokines detected in this study, supporting the concept that probiotics exert strain-specific effects on the intestinal epithelium to modulate its functionality and immune function (Kekkonen et al., 2008). Among all the probiotics, the single-strain treatments, *S. boulardii* CNCM I-1079 (SB) and *L. rhamnosus* R0011 (R0011), were consistently associated with significant changes in inflammatory cytokine production at *T* = 24 h (in 12 out of a total 16 cytokines detected). R0011 was associated with a significant (*p* < 0.05) decrease in the levels of CXCL5, TNFRSF8, IL-32, MIF, CCL21, CXCL10, CCL19, TNF-α, IL-1β, IL-6, and IFN-γ and a significant (*p* < 0.05) increase in anti-inflammatory IL-10. Similarly, *S. boulardii* CNCM I-1079 showed a significant (*p* < 0.05) decrease in the levels of IL-8, CXCL5, TNFRSF8, IL-32, MIF, CCL21, CXCL10, CCL19, TNF-α, IL-1β, and IFN-γ and a significant (*p* < 0.05) increase in anti-inflammatory IL-10. These findings indicate similar modes of immunomodulatory action for *S. boulardii* CNCM I-1079 and *L. rhamnosus* R0011 in the context of CDI. *S. boulardii* has been previously shown to inhibit IL-8 production induced by TcdA in human colonocyte NCM460 cells (Chen et al., 2006), reduce TNF-α expression caused in a hamster model of CDI (Koon et al., 2016), and exhibit immunomodulatory activity in the gut in clinical studies (Ozkan et al., 2007; Abbas et al., 2014; Consoli et al., 2016). Although *L. rhamnosus* R0011 has not been previously examined in association to CDI, studies have demonstrated its ability to downregulate IL-8 production in HT-29 epithelial cells *via* the secretion of a range of bioactive molecules (Jeffrey et al., 2018). Furthermore, in a recent study by Jeffrey et al. (2020), the secretome of *L. rhamnosus* R0011 was shown to attenuate pro-inflammatory gene expression in HT-29 cells challenged with either TNF-α or *Salmonella typhimurium* secretome (Jeffrey et al., 2020). In support of the present findings, *L. rhamnosus* R0011 secretome induced the production of MIF, leading to a downregulation of NF-κB expression, indicating that MIF exhibits a context-dependent inflammatory response to bacterial challenges (Boonma et al., 2014).

Among the rest of the probiotics, ProtecFlor™ was most effective (with a significant decrease observed at *T* = 24 h in 7/16 cytokines), followed by *L. helveticus* R0052 (6/16), *Bifidobacterium longum* R0175 (6/16), *L. rhamnosus* GG R0343 (3/16), the combination of R0011+R0052+R0175 (3/16), and the combination of R0343+SB (3/16). Interestingly, *L. rhamnosus GG* R0343, which has been demonstrated to prevent cytokine-induced apoptosis in several intestinal epithelial cell models (Yan and Polk, 2002) and to modulate serum cytokines in several clinical studies (Pohjavuori et al., 2004; Bajaj et al., 2014; Kumperscak et al., 2020), did not appear to show any major effects on T84 cell viability or CDI-FW-mediated inflammatory response. This could be due to the mode of action of *L. rhamnosus GG* R0343 that utilizes its pili to adhere onto the gut lumen, which is followed by interaction with Toll-like receptor 2 and lipoteichoic acid to modulate IL-8 mRNA expression (Lebeer et al., 2012). Thus, the absence of viable probiotic bacteria in the FW treatments could have potentially diminished the immunomodulatory ability of this probiotic on T84 cells.

This study highlights the use of a novel, high-throughput preclinical approach to characterize potential mechanisms at the gut level against *C. difficile* that have not been used previously, particularly with respect to the application of human-associated gut microbiota and multiple probiotic treatments. The use of human-associated fecal matter and microbiota is crucial to assess response to infections such as CDI, allowing for a closer representation of the *in vivo* context in comparison to animal-associated fecal matter, as the gut microbial composition and the response to CDI in animals can differ greatly from those of humans (Nguyen et al., 2015; Turner, 2018). T84 human intestinal epithelial cells were utilized to allow for a detailed assessment of the epithelial cell response, providing further information on various inflammatory biomarkers for consideration in future studies. However, due to the absence of other cell types, such as monocytes or neutrophils, the study could not comprehensively assess the effects of CDI-FW or its probiotic treatments on the adaptive and innate immune responses. Moreover, due to a lack of information regarding the genotype of the *C. difficile* strain, the study could not accurately attribute the observed effects to any strain or ribotype of interest within the North American population. In this regard, future studies with epidemic variants of *C. difficile* in animal models could provide more holistic assessments and a confirmation of the effects observed in this study, such as those with gnotobiotic mice with human fecal microbiota transplantation of CDI patients (Kumar et al., 2016).

## Conclusion

In summary, the results from our study demonstrated the ability of FW from CDI microbiota to adversely affect T84 cellular health and increase inflammatory marker production, including, for the first time, previously unreported cytokines. Specifically, exposure of T84 cells to CDI-FW caused a significant (*p* < 0.05) decrease in cell viability (Figure 1) along with increased production of several pro-inflammatory markers, including the chemokines IL-8 and CXCL5 and the cytokines TNFSRF8, IL-32, and MIF among others (Figure 2). While the roles of both IL-8 and CXCL5 in CDI pathophysiology have been previously documented (El Feghaly et al., 2013a), this study shows a potential role of TNFSRF8, IL-32, and MIF in CDI-mediated inflammation. Notably, the present study shows the ability of several probiotics to protect against CDI-FW-mediated inflammatory response. Probiotic supplementation in CDI-FW exhibited a strain-specific modulation of cellular health and inflammatory marker production, among which *S. boulardii* CNCM I-1079 and *L. rhamnosus R0011* were the most effective. In particular, these findings demonstrate that *L. rhamnosus* R0011 could play a role in modulating CDI-mediated inflammation while further elucidating the potential modes of action of *S. boulardii* in this regard. Overall, the present data support the concept that probiotic strains can modulate CDI-mediated changes in the lumen to impact upon the subsequent inflammatory response. This study also provides a novel systematic testing approach to assess probiotic efficacy in CDI involving cytokine production mediated by CDI fecal microbiota.

## Data Availability Statement

The original contributions presented in the study are included in the article/Supplementary Material, further inquiries can be directed to the corresponding author/s.

## Ethics Statement

Ethical review and approval was not required for the study on human participants in accordance with the local legislation and institutional requirements. The patients/participants provided their written informed consent to participate in this study.

## Author Contributions

MG designed the study, carried out the experiments, analyzed the data, and drafted the manuscript. SL-E helped carry out the experiments and analyze the data. SK initiated the original idea of the study, supervised all the aspects of the study, and helped review the manuscript. MI was involved in method development, data analyses, and review of the manuscript. TT contributed to the original idea of the study and its design and provided edits for the manuscript. CM helped carry out some of the experiments and provided edits for the manuscript. All authors read and approved the final manuscript.

## Conflict of Interest

The authors declare that the research was conducted in the absence of any commercial or financial relationships that could be construed as a potential conflict of interest.

## Publisher’s Note

All claims expressed in this article are solely those of the authors and do not necessarily represent those of their affiliated organizations, or those of the publisher, the editors and the reviewers. Any product that may be evaluated in this article, or claim that may be made by its manufacturer, is not guaranteed or endorsed by the publisher.
