# Supplementary material for: Probiotics Exhibit Strain-Specific Protective Effects in T84 Cells Challenged With Clostridioides difficile-Infected Fecal Water
Source: Front Microbiol. 2022 Jan 26;12:698638. doi: 10.3389/fmicb.2021.698638 (PMC8826048; doi:10.3389/fmicb.2021.698638)
Supplement: Supplementary file 1 [file Data_Sheet_1.pdf]

Supplementary Data

Table S1. Percent change of cytokine production from T84 cells exposed to CDI-FW Blank in comparison to Normal FW Blank

Production range (pg / mL)	Cytokine	% change at T = 0 h	% change at T = 24 h
0-50	**CCL19**	-40.37	-33.02
	**IL-1β**	452.85	593.33
	**IFN-γ**	52.63	17.39
	**IL-6**	-5.00	-1.69
	**CCL13**	45.45	-72.60
	**IL-10**	-35.22	-72.32
	**IL-16**	-42.41	-63.87
	**TNF-α**	149.85	298.15
51-200	**IL-11**	16.50	13.09
	**CXCL10**	37.61	106.52
201-3000	**CCL21**	31.65	13.60
	**TNFRSF8**	122.22	99.28
	**IL-8**	139.40	212.11
	**IL-32**	209.75	68.73
	**CXCL5**	154.21	104.76
10,000 +	**MIF**	167.48	**1111.29**

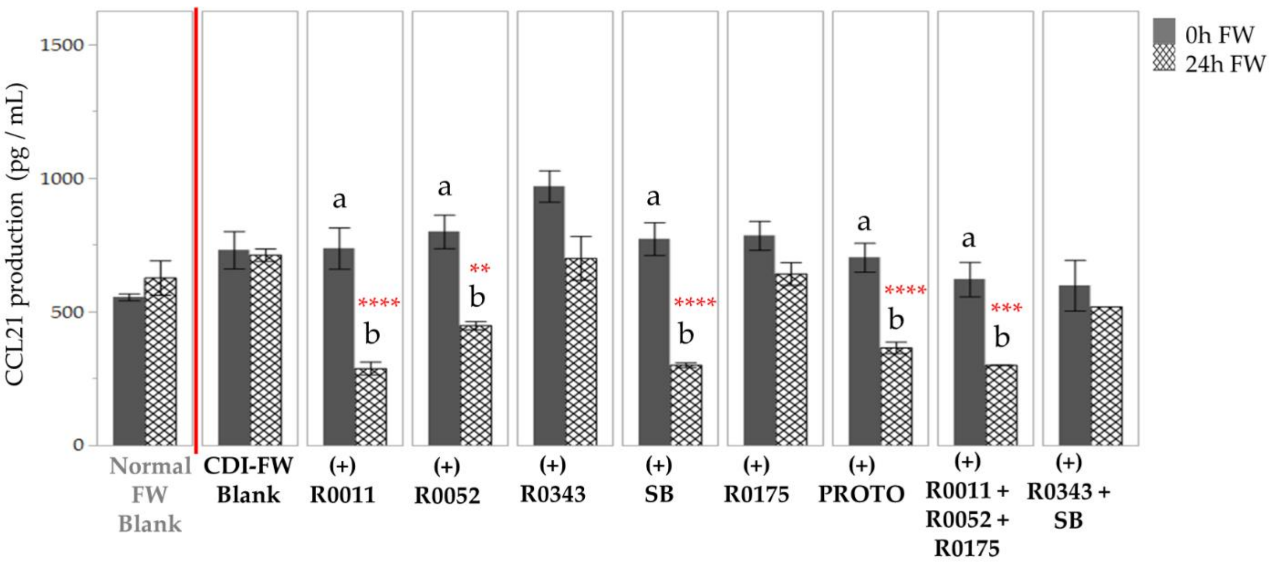

Figure S1. Detection of chemokine (C-C motif) ligand 21 (CCL21) production following exposure of T84 cells with *Clostridioides difficile*-infected (CDI) fecal water (FW) treatments as measured by multiplex assay. **(■)** cells treated with FW collected at T = 0 h, and **(▩)** cells treated FW collected at T = 24 h. Values are shown as mean ± SEM. Means at time points within treatments without a common letter are significantly different ($p < 0.05$). The symbol * represents significant differences ($p < 0.01$ = **; $p < 0.001$ = ***; $p < 0.0001$ = ****) between treatment and CDI-FW Blank at T = 24 h. R0011 = *L. rhamnosus* R0011; R0052 = *L. helveticus* R0052; R0343 = *L. rhamnosus* GG R0343; SB = *S. boulardii* CNCM I-1079; R0175 = *B. longum* R0175; PROTO = ProtecFlor^{TM}; R0011+ R0052+R0175 = combination of *L. rhamnosus* R0011, *L. helveticus* R0052 and *B. longum* R0175; R0343+SB = combination of *L. rhamnosus* GG R0343 and *S. boulardii* CNCM I-1079.

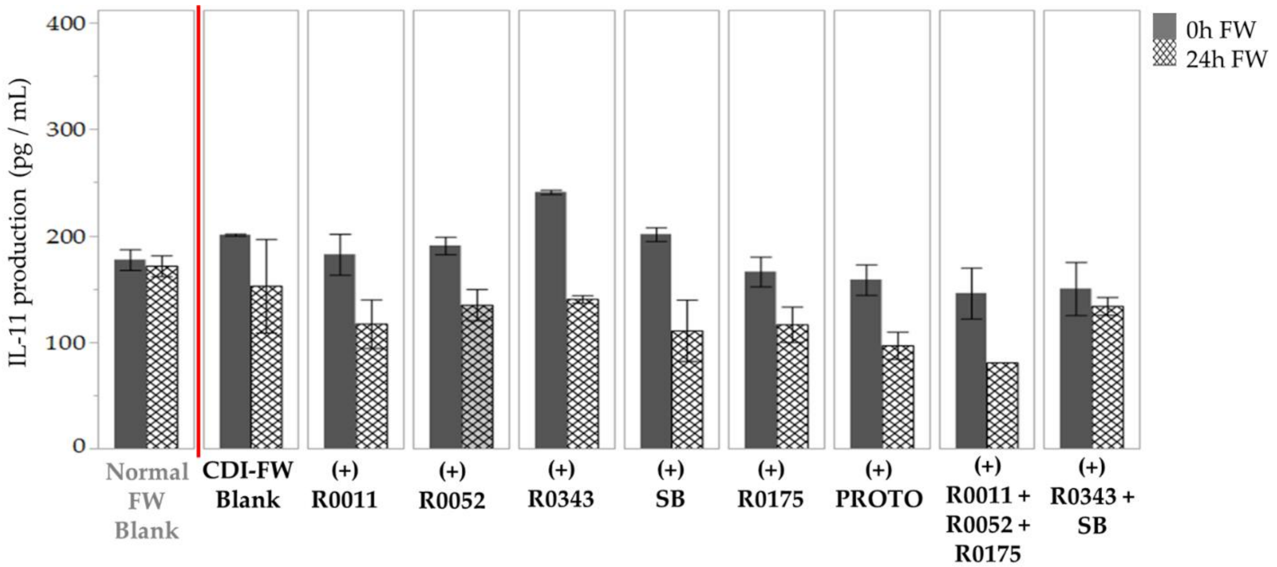

Figure S2. Detection of Interleukin-11 (IL-11) production following exposure of T84 cells with *Clostridioides difficile*-infected (CDI) fecal water (FW) treatments as measured by multiplex assay. **(■)** cells treated with FW collected at T = 0 h, and **(▩)** cells treated FW collected at T = 24 h. Values are shown as mean ± SEM. R0011 = *L. rhamnosus* R0011; R0052 = *L. helveticus* R0052; R0343 = *L. rhamnosus* GG R0343; SB = *S. boulardii* CNCM I-1079; R0175 = *B. longum* R0175; PROTO = ProtecFlor^{TM}; R0011+ R0052+R0175 = combination of *L. rhamnosus* R0011, *L.*

helveticus R0052 and *B. longum* R0175; R0343+SB = combination of *L. rhamnosus* GG R0343 and *S. boulardii* CNCM I-1079.

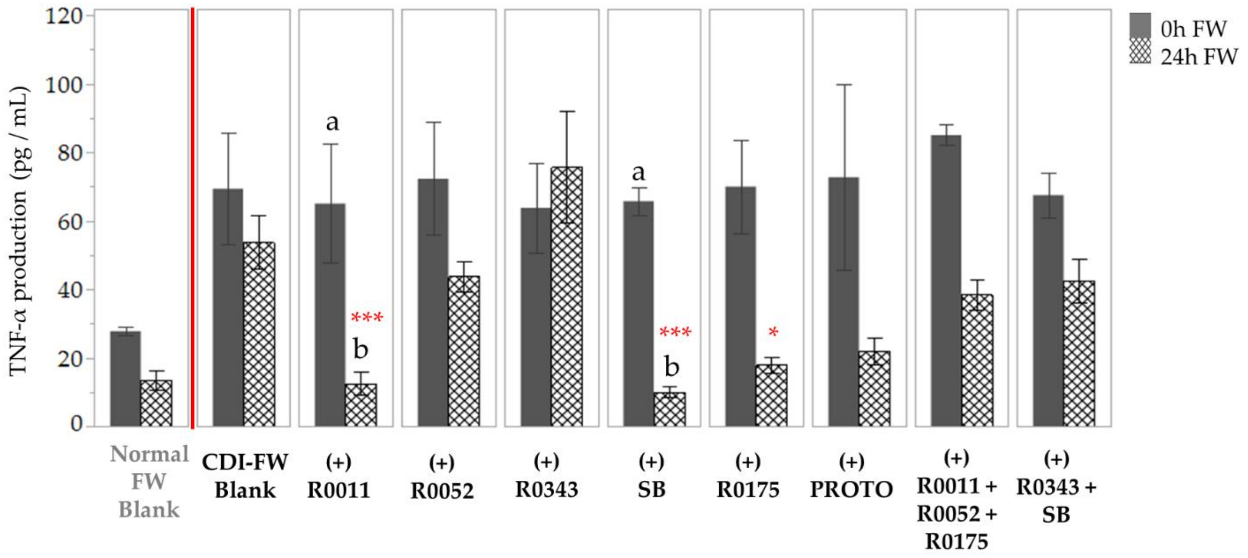

Figure S3. Detection of tumor necrosis factor (TNF) - α production following exposure of T84 cells with *Clostridioides difficile*-infected (CDI) fecal water (FW) treatments as measured by multiplex assay. **(■)** cells treated with FW collected at T = 0 h, and **(▩)** cells treated FW collected at T = 24 h. Values are shown as mean ± SEM. Means at time points within treatments without a common letter are significantly different ($p < 0.05$). The symbol * represents significant differences ($p < 0.05$ = *; $p < 0.001$ = ***) between treatment and CDI-FW Blank at T = 24 h. R0011 = *L. rhamnosus* R0011; R0052 = *L. helveticus* R0052; R0343 = *L. rhamnosus* GG R0343; SB = *S. boulardii* CNCM I-1079; R0175 = *B. longum* R0175; PROTO = ProtecFlor^{TM}; R0011+ R0052+R0175 = combination of *L. rhamnosus* R0011, *L. helveticus* R0052 and *B. longum* R0175; R0343+SB = combination of *L. rhamnosus* GG R0343 and *S. boulardii* CNCM I-1079.

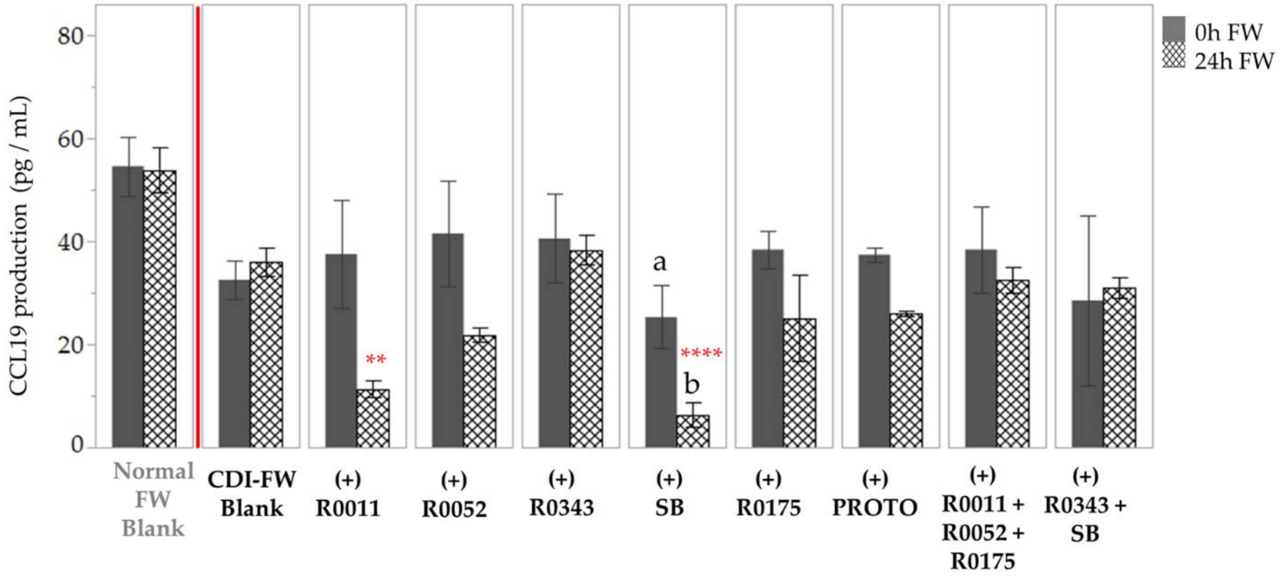

Figure S4. Chemokine (C-C motif) ligand 19 (CCL19) production following exposure of T84 cells with *Clostridioides difficile*-infected (CDI) fecal water (FW) treatments as measured by multiplex assay. **(■)** cells treated with FW collected at T = 0 h, and **(▩)** cells treated FW collected at T = 24

h. Values are shown as mean ± SEM. Means at time points within treatments without a common letter are significantly different ($p < 0.05$). The symbol * represents significant differences ($p < 0.01$ = **; $p < 0.0001$ = ****) between treatment and CDI-FW Blank at T = 24 h. R0011 = *L. rhamnosus* R0011; R0052 = *L. helveticus* R0052; R0343 = *L. rhamnosus* GG R0343; SB = *S. boulardii* CNCM I-1079; R0175 = *B. longum* R0175; PROTO = ProtecFlor™; R0011+ R0052+R0175 = combination of *L. rhamnosus* R0011, *L. helveticus* R0052 and *B. longum* R0175; R0343+SB = combination of *L. rhamnosus* GG R0343 and *S. boulardii* CNCM I-1079.

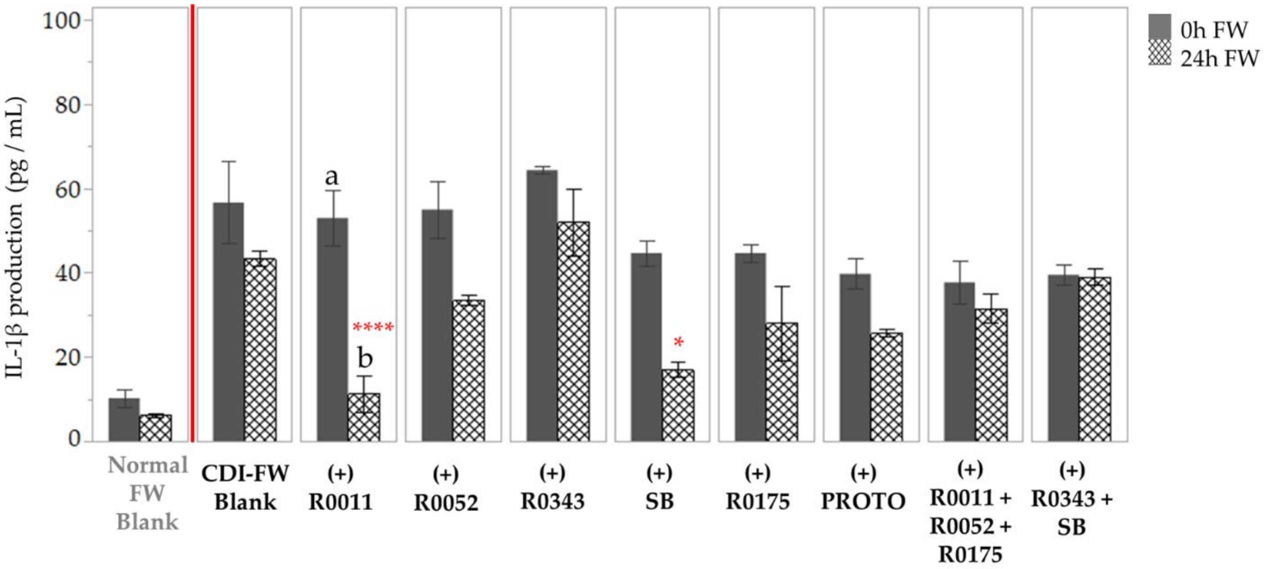

Figure S5. Interleukin (IL) -1β production following exposure of T84 cells with *Clostridioides difficile*-infected (CDI) fecal water (FW) treatments as measured by multiplex assay. **(▇)** cells treated with FW collected at T = 0 h, and **(▩)** cells treated FW collected at T = 24 h. Values are shown as mean ± SEM. Means at time points within treatments without a common letter are significantly different ($p < 0.05$). The symbol * represents significant differences ($p < 0.05$ = *; $p < 0.0001$ = ****) between treatment and CDI-FW Blank at T = 24 h. R0011 = *L. rhamnosus* R0011; R0052 = *L. helveticus* R0052; R0343 = *L. rhamnosus* GG R0343; SB = *S. boulardii* CNCM I-1079; R0175 = *B. longum* R0175; PROTO = ProtecFlor™; R0011+ R0052+R0175 = combination of *L. rhamnosus* R0011, *L. helveticus* R0052 and *B. longum* R0175; R0343+SB = combination of *L. rhamnosus* GG R0343 and *S. boulardii* CNCM I-1079.

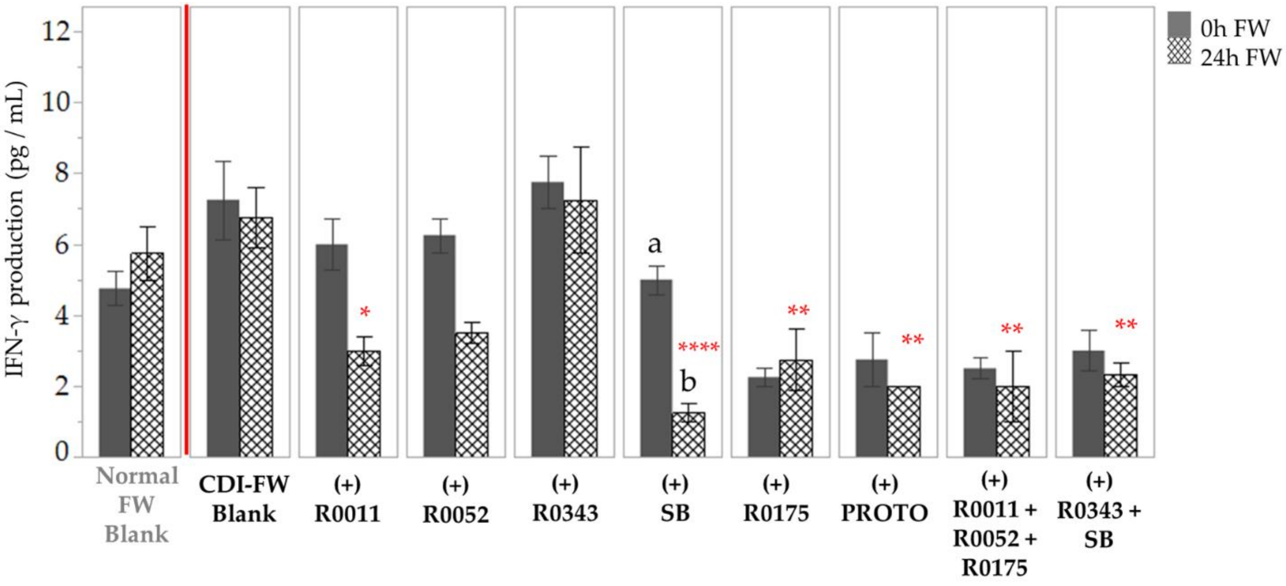

Figure S6. Interferon (IFN) -γ production following exposure of T84 cells with *Clostridioides difficile*-infected (CDI) fecal water (FW) treatments as measured by multiplex assay. **(▀)** cells treated with FW collected at T = 0 h, and **(▩)** cells treated FW collected at T = 24 h. Values are shown as mean ± SEM. Means at time points within treatments without a common letter are significantly different ($p < 0.05$). The symbol * represents significant differences ($p < 0.05$ = *; $p < 0.01$ = **; $p < 0.0001$ = ****) between treatment and CDI-FW Blank at T = 24 h. R0011 = *L. rhamnosus* R0011; R0052 = *L. helveticus* R0052; R0343 = *L. rhamnosus* GG R0343; SB = *S. boulardii* CNCM I-1079; R0175 = *B. longum* R0175; PROTO = ProtecFlor™; R0011+ R0052+R0175 = combination of *L. rhamnosus* R0011, *L. helveticus* R0052 and *B. longum* R0175; R0343+SB = combination of *L. rhamnosus* GG R0343 and *S. boulardii* CNCM I-1079.

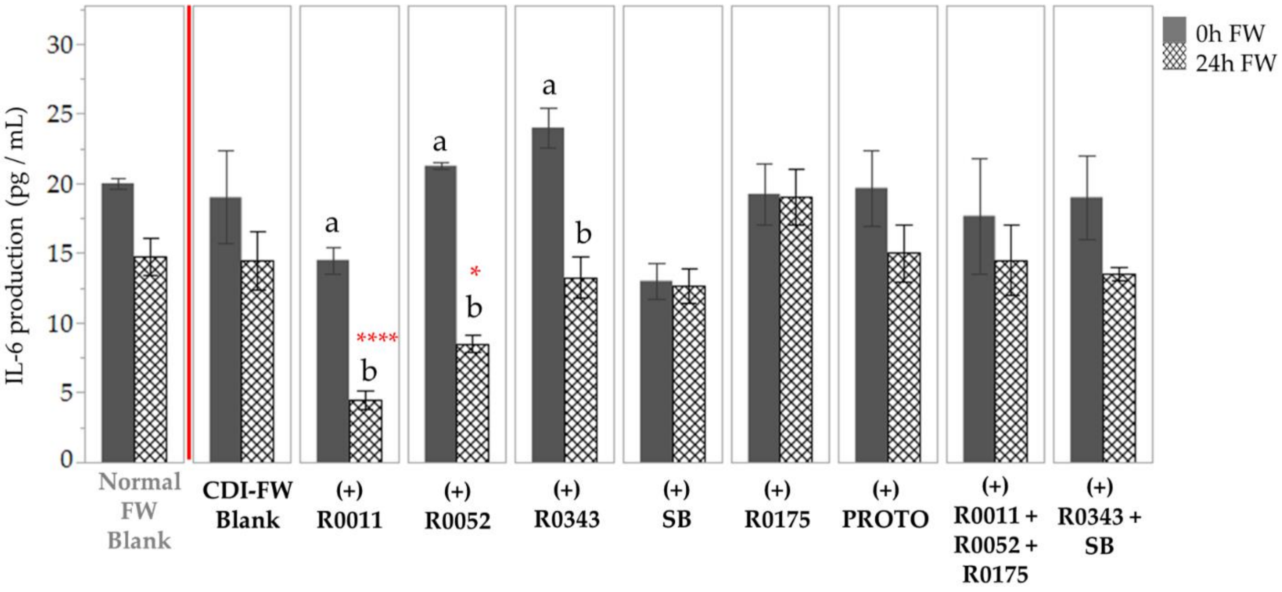

Figure S7. Interleukin (IL) -6 production following exposure of T84 cells with *Clostridioides difficile*-infected (CDI) fecal water (FW) treatments as measured by multiplex assay. **(▀)** cells treated with FW collected at T = 0 h, and **(▩)** cells treated FW collected at T = 24 h. Values are shown as mean ± SEM. Means at time points within treatments without a common letter are significantly different ($p < 0.05$). The symbol * represents significant differences ($p < 0.05$ = *; $p <$

0.0001 = ****) between treatment and CDI-FW Blank at T = 24 h. R0011 = *L. rhamnosus* R0011; R0052 = *L. helveticus* R0052; R0343 = *L. rhamnosus* GG R0343; SB = *S. boulardii* CNCM I-1079; R0175 = *B. longum* R0175; PROTO = ProtecFlor^{TM}; R0011+ R0052+R0175 = combination of *L. rhamnosus* R0011, *L. helveticus* R0052 and *B. longum* R0175; R0343+SB = combination of *L. rhamnosus* GG R0343 and *S. boulardii* CNCM I-1079.

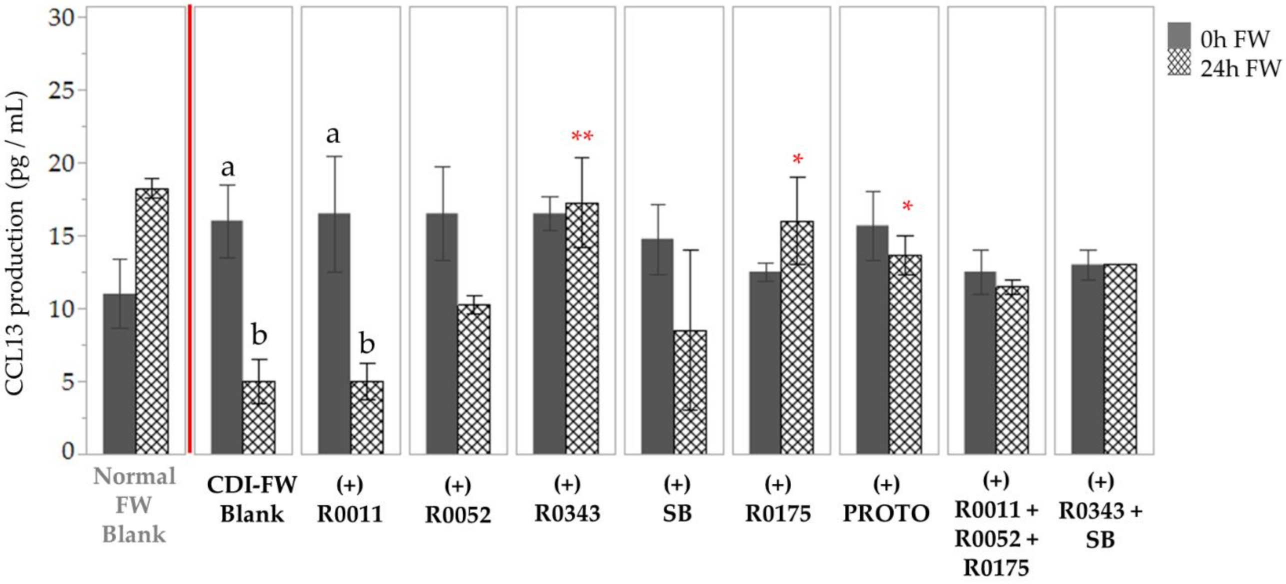

Figure S8. Chemokine (C-C motif) ligand 13 (CCL13) production following exposure of T84 cells with *Clostridioides difficile*-infected (CDI) fecal water (FW) treatments as measured by multiplex assay. **(■)** cells treated with FW collected at T = 0 h, and **(▩)** cells treated FW collected at T = 24 h. Values are shown as mean ± SEM. Means at time points within treatments without a common letter are significantly different ($p < 0.05$). The symbol * represents significant differences ($p < 0.05$ = *; $p < 0.01$ = **) between treatment and CDI-FW Blank at T = 24 h. R0011 = *L. rhamnosus* R0011; R0052 = *L. helveticus* R0052; R0343 = *L. rhamnosus* GG R0343; SB = *S. boulardii* CNCM I-1079; R0175 = *B. longum* R0175; PROTO = ProtecFlor^{TM}; R0011+ R0052+R0175 = combination of *L. rhamnosus* R0011, *L. helveticus* R0052 and *B. longum* R0175; R0343+SB = combination of *L. rhamnosus* GG R0343 and *S. boulardii* CNCM I-1079.

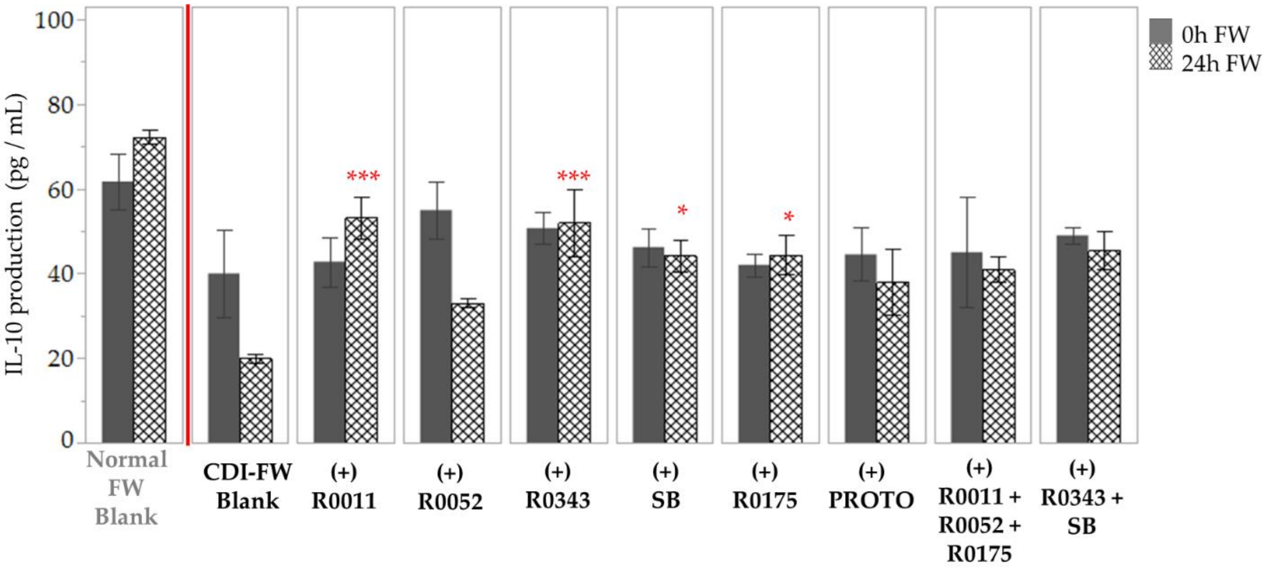

Figure S9. Interleukin (IL) -10 production following exposure of T84 cells with *Clostridioides difficile*-infected (CDI) fecal water (FW) treatments as measured by multiplex assay. **(■)** cells treated with FW collected at T = 0 h, and **(▩)** cells treated FW collected at T = 24 h. Values are shown as mean ± SEM. The symbol * represents significant differences ($p < 0.05$ = *; $p < 0.001$ = ***) between treatment and CDI-FW Blank at T = 24 h. R0011 = *L. rhamnosus* R0011; R0052 = *L. helveticus* R0052; R0343 = *L. rhamnosus* GG R0343; SB = *S. boulardii* CNCM I-1079; R0175 = *B. longum* R0175; PROTO = ProtecFlor^{TM}; R0011+ R0052+R0175 = combination of *L. rhamnosus* R0011, *L. helveticus* R0052 and *B. longum* R0175; R0343+SB = combination of *L. rhamnosus* GG R0343 and *S. boulardii* CNCM I-1079.

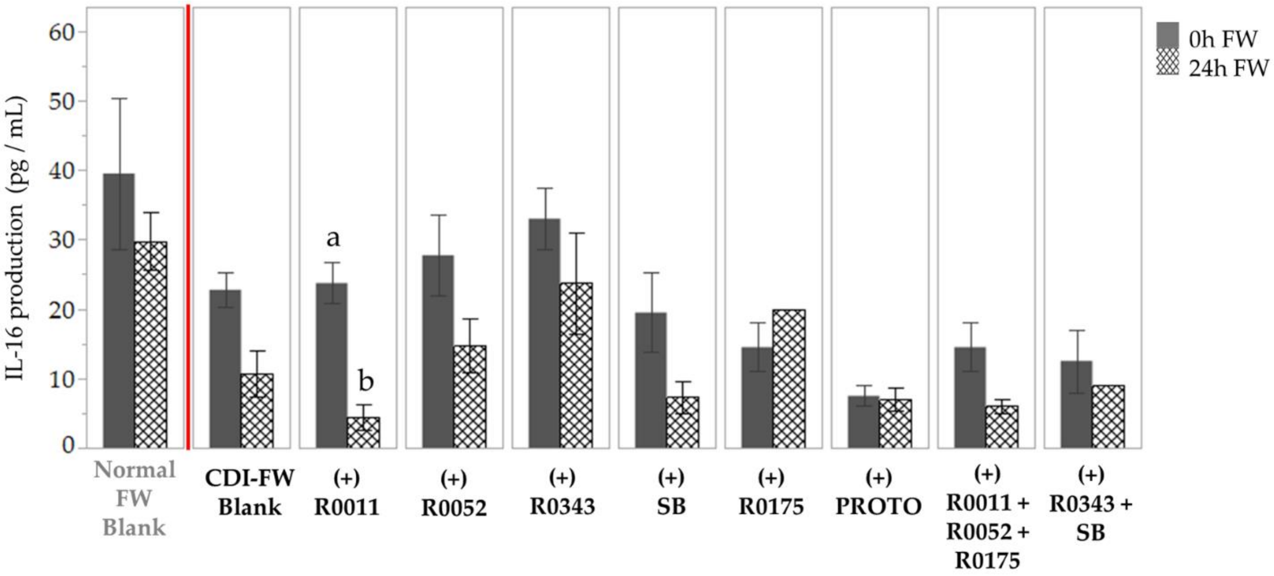

Figure S10. Interleukin (IL) -16 production following exposure of T84 cells with *Clostridioides difficile*-infected (CDI) fecal water (FW) treatments as measured by multiplex assay. **(■)** cells treated with FW collected at T = 0 h, and **(▩)** cells treated FW collected at T = 24 h. Values are shown as mean ± SEM. Means at time points within treatments without a common letter are significantly different ($p < 0.05$). R0011 = *L. rhamnosus* R0011; R0052 = *L. helveticus* R0052; R0343 = *L. rhamnosus* GG R0343; SB = *S. boulardii* CNCM I-1079; R0175 = *B. longum* R0175; PROTO = ProtecFlor^{TM}; R0011+ R0052+R0175 = combination of *L. rhamnosus* R0011, *L. helveticus* R0052 and *B. longum* R0175; R0343+SB = combination of *L. rhamnosus* GG R0343 and *S. boulardii* CNCM I-1079.

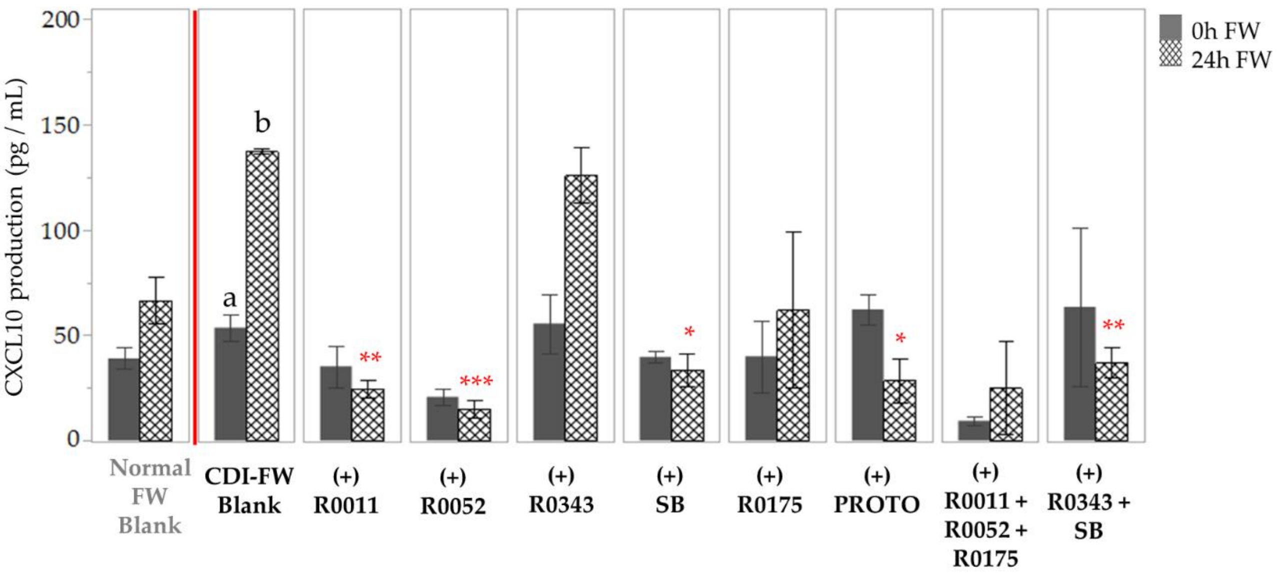

Figure S11. C-X-C motif chemokine ligand 10 (CXCL10) production following exposure of T84 cells with *Clostridioides difficile*-infected (CDI) fecal water (FW) treatments as measured by multiplex assay. **(■)** cells treated with FW collected at T = 0 h, and **(▩)** cells treated FW collected at T = 24 h. Values are shown as mean ± SEM. Means at time points within treatments without a common letter are significantly different ($p < 0.05$). The symbol * represents significant differences ($p < 0.05$ = *; $p < 0.01$ = **; $p < 0.001$ = ***) between treatment and CDI-FW Blank at T = 24 h. R0011 = *L. rhamnosus* R0011; R0052 = *L. helveticus* R0052; R0343 = *L. rhamnosus* GG R0343; SB = *S. boulardii* CNCM I-1079; R0175 = *B. longum* R0175; PROTO = ProtecFlor^{TM}; R0011+ R0052+R0175 = combination of *L. rhamnosus* R0011, *L. helveticus* R0052 and *B. longum* R0175; R0343+SB = combination of *L. rhamnosus* GG R0343 and *S. boulardii* CNCM I-1079.
